# Impact of polycystic ovary syndrome on quality of life of women in correlation to age, basal metabolic index, education and marriage

**DOI:** 10.1371/journal.pone.0247486

**Published:** 2021-03-10

**Authors:** Fauzia Tabassum, Chandra Jyoti, Hemali Heidi Sinha, Kavita Dhar, Md Sayeed Akhtar

**Affiliations:** 1 Department of Pharmacology, Santosh Medical College, Santosh University, Ghaziabad, Uttar-Pradesh, India; 2 Department of Gynecology and Obstetrics, All India Institute of Medical Sciences, Patna, Bihar, India; 3 College of Pharmacy, King Khalid University, Abha, Kingdom of Saudi Arabia; University of Insubria, ITALY

## Abstract

Polycystic ovary syndrome (PCOS) is the major endocrine related disorder in young age women. Physical appearance, menstrual irregularity as well as infertility are considered as a sole cause of mental distress affecting health-related quality of life (HRQOL). This prospective case-control study was conducted among 100 PCOS and 200 healthy control cases attending tertiary care set up of AIIMS, Patna during year 2017 and 2018. Pre-validated questionnaires like Short Form Health survey-36 were used for evaluating impact of PCOS in women. Multivariate analysis was applied for statistical analysis. In PCOS cases, socioeconomic status was comparable in comparison to healthy control. But, PCOS cases showed significantly decreased HRQOL. The higher age of menarche, irregular/delayed menstrual history, absence of child, were significantly altered in PCOS cases than control. Number of child, frequency of pregnancy, and miscarriage were also observed higher in PCOS cases. Furthermore, in various category of age, BMI, educational status and marital status, significant differences were observed in the different domain of SF-36 between PCOS and healthy control. Altogether, increased BMI, menstrual irregularities, educational status and marital status play a major role in altering HRQOL in PCOS cases and psychological care must be given during patient care.

## Introduction

Polycystic ovary syndrome (PCOS) is a major endocrine disorder in young age women affecting their health-related quality of life (HRQOL) and their mental well-being as well [1,2]. Moreover, this develop into lifelong health condition that continues far beyond the young ages and affects around 5 million young age population in the United States of America [3,4]. In India, PCOS has been reported to vary between racial counterparts with an estimated prevalence of 9.13% in adolescents [5]. The major changes in physical appearance, obesity, along with menstrual irregularity have been found to be the main contributing factor of psychological dilemma [6–8]. PCOS negative impact is always underestimated and dominates on women’s life and may lead to a risk for serious anxiety and psychological disorder [9,10]. Importantly, the psychological burden greatly varies with the change in geographical areas and societal perceptions (Barnard et al., 2007; Brady et al., 2009). These patients may experience characteristics of PCOS as stressful and may be at higher risk for depression and anxiety disorders and even this may lead towards suicidal tendency [9,10].

Clinically, PCOS is characterized by either oligoovulation or anovulation and hyperandrogenism that may cause infertility, and other related metabolic disorders [11]. This progresses to increased risk of reproductive issues like infertility endometrial cancer, gestational as well as mental disturbances [12]. However, novel treatments and therapies can then be targeted toward improving those problems, which are most important for the individual concerned [13,14]. Recently, increased importance has been given on understanding the impact of PCOS symptoms and in particular about the feminine identity and thus their treatment from the patients’ perspective for the better quality of life (QOL). HRQOL is a self-perceived health status as a consequence of any disease that is measured by health status questionnaires [15]. Therefore, HRQOL questionnaires like Short Form Health Survey-36 (SF-36) for PCOS, was used to understand the impact of PCOS and evaluating individual patients’ health status and monitoring and comparing disease burden [16,17]. The SF-36 scale leaves out important detrimental issues linked to PCOS patients such as physical and emotional symptoms associated with menses [16]. PCOS questionnaire has reasonable internal reliability, good test-retest reliability, good concurrent and discriminated validity, and a reasonable factor analysis making PCOS questionnaire a useful and promising tool for HRQOL in PCOS cases.

At present, there is a paucity of information related to PCOS among women of the reproductive age group in India, in particular, North India. Thus, considering these factors into account, this prospective study was planned to compare socioeconomic status (SDS) and association of age, body mass index (BMI), education level and marital status between PCOS and healthy control cases among the women in the reproductive age group visiting the department of gynaecology and obstetrics of tertiary care hospital.

## Material and methods

### Ethical approval

Ethical approval (SU/2017/1226-3) was obtained from the institutional review board of Santosh medical college, Uttar Pradesh, India. The institutional review board of All India Institute of Medical Sciences, Patna, India, granted study site approval (176/AIIMS/PAT/IEC/2017). Informed consent form was obtained from parents or guardians of the minors (<18 years).

### Study design

This prospective, cross sectional, observational study was designed and conducted in the tertiary care teaching hospital of north India.

### Study setting

Patients visiting the outpatients’ department of Gynecology and Obstetrics, All India Institute of Medical Sciences, Patna (India) were included in the study.

### Participants

Patients diagnosed with PCOS, based on criteria derived from the 2003 ESHRE/ASRM (Rotterdam criteria) were arbitrarily enrolled in the study. PCOS is diagnosed as the presence of at least two of three of the following: 1) Oligo/anovulation, 2) hyperandrogenism, 3) Polycystic ovaries [18]. A healthy control (HC) was selected from participants of the same population and having regular menses and had no clinical features of hyperandrogenism as well as infertility.

### Data sources

Data was collected after describing both written and verbal information about the study. After explaining, the informed consent form was signed by each participant and then they were requested to complete the questionnaires. Face-to-face interviews were conducted by investigators to the subjects meeting the inclusion criteria and consented for the participation into the study in three parts: Part A: Semi-structured, pre-validated questionnaires were used for collecting information on the socio-demographic, economic and reproductive history. Part B: Pre-validated SF-36 questionnaire is a standard diagnostic tool for evaluating various aspects of the HRQOL over the previous 4 weeks [19]. Its validity, sensitivity, reliability, internal consistency and stability, as well as test-retest reliability have frequently been confirmed in various studies [20–22]. SF-36 contains 8 domains: general health, physical functioning, and role limitations due to physical health, role limitation due to the emotional problem, body pain, social functioning, energy/fatigue and emotional well-being. The scores for each domain range from 0–100, where higher scores indicate better condition.

### Study size

The sample size was estimated post assuming α-error of 0.05, power of 80%, percentage of controls having a poor quality of life to be 20% based on previous studies and odds of poor QOL among cases to be twice than among controls. Hence, a total of 100 PCOS cases and 200 healthy control cases were enrolled in the study.

### Inclusion criteria

We included all diagnosed case of PCOS only, female from menarche to menopausal age between the age of 10–49 years, and those given informed consent.

### Exclusion criteria

Patients having cognitive or developmental disabilities/another major illness that substantially influenced the HRQOL of women, confirmed malignancy and deformities, as well as breastfeeding women were excluded from the study.

### Statistical analysis

Data were analyzed by using statistical software-Stata Version 14.0 (Stata Corp, Texas, USA). After checking for the normality condition for continuous variables, the appropriate statistical test was applied. Confounders like excessive body weight were taken into consideration. Quantitative data expressed as mean±SD, minimum and maximum followed normal and skewed distribution respectively. Analysis of covariance model (ANCOVA) was used to address potential confounders. Categorical variables expressed as frequency and percentage. Pearson Chi-Square test and Fisher exact test were used to checking the association between qualitative variables and categorical variables. Logistic regression analysis was used to estimate odds (95% CI) and models were robust for PCOS and other variables. Multivariable linear regression analysis was performed to observe the association between the variables. Independent t-test and One Way ANOVA used to compare normally distributed continuous variable between two and three categories respectively. Rank sum/Kruskal Wallis test used for comparing skewed continuous variables among categories and to look association between demographic categories. For all statistical tests, P-value < 0.05 is considered as statistical significant.

## Result

The outcome of socioeconomic status (SES) of a woman with PCOS and HC cases are mentioned in Table 1. The women with PCOS and HC were comparable in respect of marital status and family type. Statistically significant differences were observed between PCOS and HC in terms of age (P<0.020), BMI (P<0.001), educational status (P<0.001), marital status (P>0.05) and work category (P<0.001). Total 97% of PCOS case was below the age of 30 years in comparison to 78% of control. Among all PCOS cases, 60% was student and almost 54% received higher education. Among the HC group, 39% was student and only 15% received higher education (P<0.001). A higher percentage of PCOS cases (16%) belong to greater BMI (>30) in comparison to HC (2%).

**Table 1:** Comparison between sociodemographic status of PCOS and HC cases.

Age in years	PCOS n (%)	HC n (%)	Pr	Odd ratio 95% CI
≤19	34(34.0)	55 (27.5)	0.020	1
20–30	57(57.0)	101 (50.5)		0.912(0.533–1.561)
>30	9(9.0)	44 (22.0)		0.330(0.143–0.76)
**BMI**	**PCOS n (%)**	**HC n (%)**	**Pr**	**Odd ratio 95% CI**
<18.5	10(10.0)	2 (1.0)	0.000	1
18.5-<25	48(48.0)	138 (69.0)		0.069(0.014–0.328)
25-<30	26(26.0)	56(28.0)		0.092(0.018–0.454)
≥30	16(16.0)	4 (2.0)		
				0.800(0.123–5.20)
**Variable**	**PCOS n (%)**	**HC n (%)**	**Pr**	**Odd ratio 95% CI**
Illiterate	4(4.0)	14 (7.0)	0.0001	1
Up to Primary	7(7.0)	10 (5.0)		2.45(0.56–10.6)
Up to Middle	7(7.0)	25 (12.50)		0.98(0.24–3.94)
Up to High School	13(13.0)	58 (29.0)		0.78(0.22–2.77)
Intermediate	15(15.0)	63 (31.50)		0.83(0.23–2.89)
Graduation/Above	54(54.0)	30 (15.0)		6.3(1.90–20.86)
**Variable**	**PCOS n (%)**	**HC n (%)**	**Pr**	**Odd ratio 95% CI**
Married	41(41.0)	105 (52.5)	0.060	1
Unmarried	59(59.0)	95 (47.5)		1.59 (0.97–2.58)
**Variables**	**PCOS n (%)**	**HC n (%)**	**Pr**	**Odd ratio 95% CI**
Students	60(60.0)	79 (39.5)	0.000	1
House maker	35(35.0)	98 (49)		0.470(0.281–0.78)
Unemployed	2(2.0)	0 (0.0)		0.171(0.049–0.59)
Employed/Professionals	3(3.0)	23 (11.5)		0.759(0.54–1.06)

PCOS: Polycystic ovary syndrome; HC: Healthy control; BMI: Body mass index; Pr: Predictive value; CI: Confidence interval; n: Number of patients; %: Percentage.

As shown in Table 2, among the PCOS group, a significant percentage of women (33%) has menarche at age greater than 14 years and there was no any HC cases lies in this category (P<0.001). In respect of menstrual history, PCOS cases have a higher percentage of irregular (45%) and delayed (54%) menses and this comprises a signify`cant difference (P<0.001) in comparison to HC that was 8% irregular and no any delay in menses were observed. Around 64.3% of cases of PCOS women have no child (P<0.001) in contrast to HC cases (9.5%). However, 86.67% PCOS cases have less than ≤ 2 children in comparison to HC where 69.47% have less than ≤2 children (P<0.169). In terms of pregnancy, almost 77.27% PCOS women got pregnant ≤2 times in comparison to HC cases (P>0.05).

**Table 2:** Comparison of menstrual and reproductive history of women with PCOS and HC cases.

Menstrual and reproductive history					
Variables	Groups	PCOS n (%)	HC n (%)	Pr	Odd ratio 95% CI
**Age at menarche**	11–14	67(67.0)	200(100.0)	0.000	1
	>14	33(33.0)	0(00.0)		
**Menstrual history**	Regular	1(1.0)	192(96.0)	0.000	1
	Irregular	45(45.0)	8(4.0)		1079.99 (131.71–8855.21)
	Delayed/Late	54(54.0)	0(0.0)		
**Children**	Yes	15(35.7)	95(90.5)	0.000	1
	No	27(64.3)	10(9.5)		17.1 (6.90–42.36)
**Number of children**	≤2	13(86.67)	66(69.47)	0.169	1
	>2	2(13.33)	29(30.53)		0.350 (0.074–1.65)
**How many times pregnant**	≤2	17(77.27)	58(61.05)	0.153	1
					0.461(0.156–1.35)
	>2	5(22.73)	37(38.95)		
	No	88(88.0)	185(92.5)		

PCOS: Polycystic ovary syndrome; HC: Healthy control case; Pr: Predictive value; CI: Confidence interval; n: Number of patients; %: Percentage.

As depicted in Table 3, Overall differences of mean in PCOS and HC case comparable in respect of BMI (P<0.125).

**Table 3:** Comparison of different variables between PCOS and HC cases.

Variables	PCOS mean± SD	p50(min-max)	HC mean± SD	*P-value*
**Age**	22.81±5.33	22(14–36)	24.67±6.04	0.009
**BMI**	24.35±5.49	24.29(11.32–38.1)	23.61±2.79	0.125
**Age at marriage**	19.31±3.97	20(13–30)	22.29±2.36	0.000
**Age at menarche**	13.86±1.44	14(11–18)	12.64±1.09	0.000
**No of child**	1.46±0.91	1(1–4)	2.26±1.04	0.000
**How many times pregnant**	1.77±1.02	1(1–4)	2.49±1.36	0.006

Data are presented as mean± SD; P value less than 0.05 is considered as significant differences between the group; PCOS: Polycystic ovary syndrome; HC: Healthy control; BMI: Body mass index; P50: Probability 50 value; SD: Standard deviation.

However, the highest range of BMI was more in PCOS cases in comparison to HC. Whereas, a statistically, significant differences in mean were observed in respect of age (P<0.009), age at marriage (P<0.001), age of menarche (P<0.001), number of children (P<0.001) and number of pregnancy (P<0.006) between PCOS and HC cases. In case of age, women with PCOS at age ≤19 showed significantly higher score for of general health (P<0.001), role limitation due to physical health (P<0.001), role limitation due to the emotional problem (P<0.022), pain (P<0.025) and social function (P<0.010) in comparison to age >30. However, comparable differences were observed in physical function (P<0.116), energy or fatigue (P<0.087) and emotional well-being (P<0.108). In HC cases, women of age ≤19 showed a statistically higher score in general health (P<0.001), physical health (P<0.001), role limitation due to the emotional problem (P<0.005) and energy/fatigue (P<0.001). Comparable differences were observed for role limitation due to physical health (P<0.818), pain (P<0.424), social functioning (P<0.110) and emotional well-being (P<0.147; Table 4).

**Table 4:** Comparison of mean score of SF-36 across various age groups in PCOS and control cases.

*Domain (PCOS)*	Age ≤19	Age ≤20–30	Age >30	*P-Value*
**General Health**	43.50±8.34	39.18±10.79	28.24±9.02	0.000
**Physical function**	84.70±20.48	75.35±21.60	75.55±19.27	0.116
**Role limitations due to physical health**	63.23±35.48	51.31±40.75	25.0±33.07	0.030
**Role limitations due to emotional problem**	64.70±38.43	53.21±36.65	25.92±36.49	0.022
**Pain**	68.16±18.72	61.71±14.42	52.22±18.04	0.025
**Social function**	68.75±16.06	60.96±15.32	52.77±11.72	0.010
**Energy/Fatigue**	57.35±12.56	52.71±10.09	50.00±11.72	0.087
**Emotional well-being**	67.41±14.57	61.47±13.68	59.55±13.63	0.108
***Domain (HC)***	**Age≤19**	**Age≤20–30**	**Age >30**	***P-Value***
**General Health**	71.36K±7.69	68.72±9.74	63.35±10.18	0.000
**Physical function**	100.0±0.000	98.96±3.48	96.47±6.69	0.000
**Role limitations due to physical health**	99.55 ±3.37	99.26±7.46	98.86±7.53	0.878
**Role limitations due to emotional problem**	100.0±0.000	100.0±0.000	94.69±20.26	0.005
**Pain**	98.68±5.01	97.42±7.32	97.21±5.63	0.424
**Social function**	99.54±3.37	99.13±3.19	97.72±7.27	0.110
**Energy/Fatigue**	91.27±5.71	88.56±7.32	86.02±7.11	0.001
**Emotional well-being**	90.83±7.12	92.33±3.14	91.09±3.64	0.147

Data are presented as mean± SD; P value less than 0.05 is considered as significant differences between the group; PCOS: Polycystic ovary syndrome; HC: Healthy control; SD: Standard deviation.

As per BMI is concerned, PCOS women scored high and statistically significant differences were observed in case of BMI those who have value <18 in comparison to >30. In addition, significant differences were observed for general health score (P<0.001), physical health (P<0.001), energy and emotion (P<0.001). Whereas, comparable differences observed in role limitation due to physical health (P<0.085), role limitation due to the emotional problem (P<0.565), pain (P<0.189), social function (P<0.549) and emotional well-being (P<0.127). In HC case, there no significant difference was observed in all the eight domains of SF-36 (Table 5). As per the level of education is concerned, HRQOL score was higher in all eight domain of SF-36 in well-educated women in comparison to illiterate or women having education of primary level, but this difference was observed to be statistically non-significant and comparable. In case of HC women, HRQOL score in graduation level was higher and significant differences were observed in relation to the level of education for SF-36 domains like general health (P<0.001), physical health (P<0.039) and energy/fatigue (P<0.003). However, we observed comparable differences among all other domains of SF-36 (Table 6).

**Table 5:** Comparison of mean score of SF-36 across various BMI groups in PCOS and HC cases.

*Domain (PCOS)*	BMI less than 18.5	BMI 18.5–24.9	BMI 25–29.9	BMI 30 and above	*P-value*
**General Health**	45.00±4.30	42.70±10.11	36.37±11.15	32.55±9.15	0.001
**Physical function**	89.00±10.48	87.08±14.76	69.23±21.00	61.56±27.30	0.000
**Role limitations due to physical health**	52.5±32.16	62.5±39.27	46.15±38.53	35.93±40.79	0.085
**Role limitations due to emotional problem**	50.00±36.0	59.02±37.18	55.12±37.64	43.75±45.08	0.576
**Pain**	68.75±14.9	65.02±15.38	60.86±17.73	56.56±19.21	0.189
**Social function**	62.5±15.95	64.58±16.77	62.98±15.60	57.81±15.05	0.549
**Energy/Fatigue**	61.0±7.74	56.87±11.28	49.61±9.37	48.43±11.65	0.001
**Emotional well-being**	67.20±13.83	65.91±12.75	58.76±13.32	60.50±18.11	0.127
***Domain (HC)***	**BMI less than 18.5**	**BMI 18.5–24.9**	**BMI 25–29.9**	**BMI 30 and above**	***P-value***
**General Health**	68.75±2.94	68.62±9.06	67.11±11.02	71.87±14.97	0.676
**Physical function**	100.0±0.00	99.05±3.22	97.67±5.95	100.00±0.00	0.175
**Role limitations due to physical health**	100.0±0.00	98.91±7.91	100.0±0.00	100.0±0.00	0.764
**Role limitations due to emotional problem**	100.0±0.00	99.51±5.67	97.02±15.92	100.00±0.00	0.437
**Pain**	100.0±0.00	97.86±5.65	97.14±8.23	100.00±0.00	0.736
**Social function**	100.0±0.00	98.91±4.64	98.88±4.31	100.00±0.00	0.951
**Energy/Fatigue**	87.50±3.53	89.27±7.00	87.41±7.44	90.00±4.08	0.402
**Emotional well-being**	92.00±0.00	91.47±5.08	91.78±3.69	93.00±5.03	0.909

Data are presented as mean± SD; P value less than 0.05 is considered as significant differences between the group; PCOS: Polycystic ovary syndrome; HC: Healthy control; BMI: Body mass index; SD: Standard deviation.

**Table 6:** Comparison of mean score of SF-36 across various educational levels in PCOS and HC cases.

**Domain (PCOS)**	**Illiterate**	**Up to primary**	**Up to middle**	**Up to high school**	**Intermediate**	**Graduation and above**	**P-Value**
**General Health**	33.35±16.31	34.10±10.93	40.47±13.10	35.56±13.56	40.01±8.74	41.59±9.22	0.234
**Physical function**	62.5±23.97	81.42±23.57	76.42±23.04	78.46±25.93	79.33±19.71	79.44±20.36	0.773
**Role limitations due to physical health**	12.50±25.0	50.00±43.30	42.85±37.40	59.61±40.23	50.0±36.59	56.94±40.16	0.327
**Role limitations due to emotional problem**	25.00±31.92	47.61±32.54	23.8±37.08	48.72±44.32	57.77±40.76	62.34±35.50	0.085
**Pain**	63.75±10.89	52.5±12.41	57.5±21.79	63.07±24.43	65.33±16.71	64.44±14.71	0.531
**Social function**	46.87±11.96	62.5±10.20	66.07±9.44	62.5±23.93	66.66±14.68	62.73±15.45	0.408
**Energy/Fatigue**	46.25±8.53	50.00±7.07	56.43±11.44	52.69±14.37	58.67±10.43	53.89±11.14	0.328
**Emotional well-being**	49.00±10.96	62.29±11.96	60.57±13.746	60.62±19.51	66.40±11.39	64.67±13.69	0.303
**Domain (HC)**	**Illiterate**	**Up to primary**	**Up to middle**	**Up to high school**	**Intermediate**	**Graduation and above**	**P-Value**
**General Health**	59.82±10.15	67.08±9.69	64.34±9.33	68.90±7.96	68.38±9.27	74.43±10.13	0.000
**Physical function**	96.43±9.28	99.50±1.58	97.40±4.59	99.66±1.81	98.33±4.57	99.50±2.01	0.039
**Role limitations due to physical health**	100.00±00	100.00±00	100.00±00	100.00±00	99.60±3.15	95.83±16.19	0.084
**Role limitations due to emotional problem**	100.00±00	100.00±00	96.00±20.0	98.85±8.75	100.00±00	97.77±12.12	0.588
**Pain**	95.53±7.97	97.00±4.83	95.70±12.19	99.13±2.83	97.85±5.78	97.66±5.04	0.212
**Social function**	97.32±7.23	100.00±00	98.50±4.14	98.70±4.49	99.60±2.20	98.75±6.84	0.522
**Energy/Fatigue**	83.21±9.32	88.00±5.37	85.80±6.40	89.14±6.63	90.08±6.12	90.50±8.13	0.003
**Emotional well-being**	92.00±1.5	90.80±4.63	91.52±4.05	90.69±6.50	91.68±3.46	93.33±3.97	0.245

Data are presented as mean± SD; P value less than 0.05 is considered as significant differences between the group; PCOS: Polycystic ovary syndrome; HC: Healthy control; SD: Standard deviation.

We observed significant differences between married and unmarried PCOS cases in terms of general health (P<0.001), physical functioning (P<0.027), role limitation due to physical health (P<0.006), role limitations due to emotional problems (P<0.002), pain (P<0.001), social functioning (P<0.001), energy/fatigue (P<0.003) and emotional well-being (P<0.001). Whereas, in HC cases, no differences were observed between married and unmarried cases in regarding SF-36 domain score for role limitation due to physical health (P<0.538), role limitation due to the emotional problem (P<0.105), Pain (P<0.044), social functioning (P<0.225), emotional well-being (P<0.857). However, significant differences were observed for general health (P<0.001), physical health (P<0.002) and energy/fatigue (P<0.001) among married and unmarried HC cases (Table 7).

**Table 7:** Comparison of mean score of SF-36 verses marital status groups in PCOS and HC cases.

Domain (PCOS)	Married	Single	P-Value
**General Health**	34.55±11.64	43.22±8.22	0.000
**Physical function**	72.92±22.24	82.45±19.87	0.027
**Role limitations due to physical health**	40.24±39.48	61.86±37.24	0.006
**Role limitations due to emotional problem**	40.65±36.90	64.40±36.54	0.002
**Pain**	56.58±15.29	67.54±16.40	0.001
**Social function**	56.09±13.72	67.58±15.93	0.001
**Energy/Fatigue**	50.12±9.84	56.78±11.51	0.003
**Emotional well-being**	57.56±13.29	67.32±13.45	0.001
**Domain (HC)**	**Married**	**Single**	**P-Value**
**General Health**	64.88±9.63	72.01±8.37	0.000
**Physical function**	97.85±5.08	99.63±2.55	0.002
**Role limitations due to physical health**	99.52±4.88	98.95±8.08	0.538
**Role limitations due to emotional problem**	97.77±13.29	100.00±0.00	0.105
**Pain**	96.85±7.53	98.68±4.72	0.044
**Social function**	98.57±5.29	99.34±3.34	0.225
**Energy/Fatigue**	86.04±7.06	91.73±5.82	0.000
**Emotional well-being**	91.65±3.14	91.53±5.97	0.857

Data are presented as mean± SD; P value less than 0.05 is considered as significant differences between the group; PCOS: Polycystic ovary syndrome; HC: Healthy control; SD: Standard deviation.

**Fig 1:**
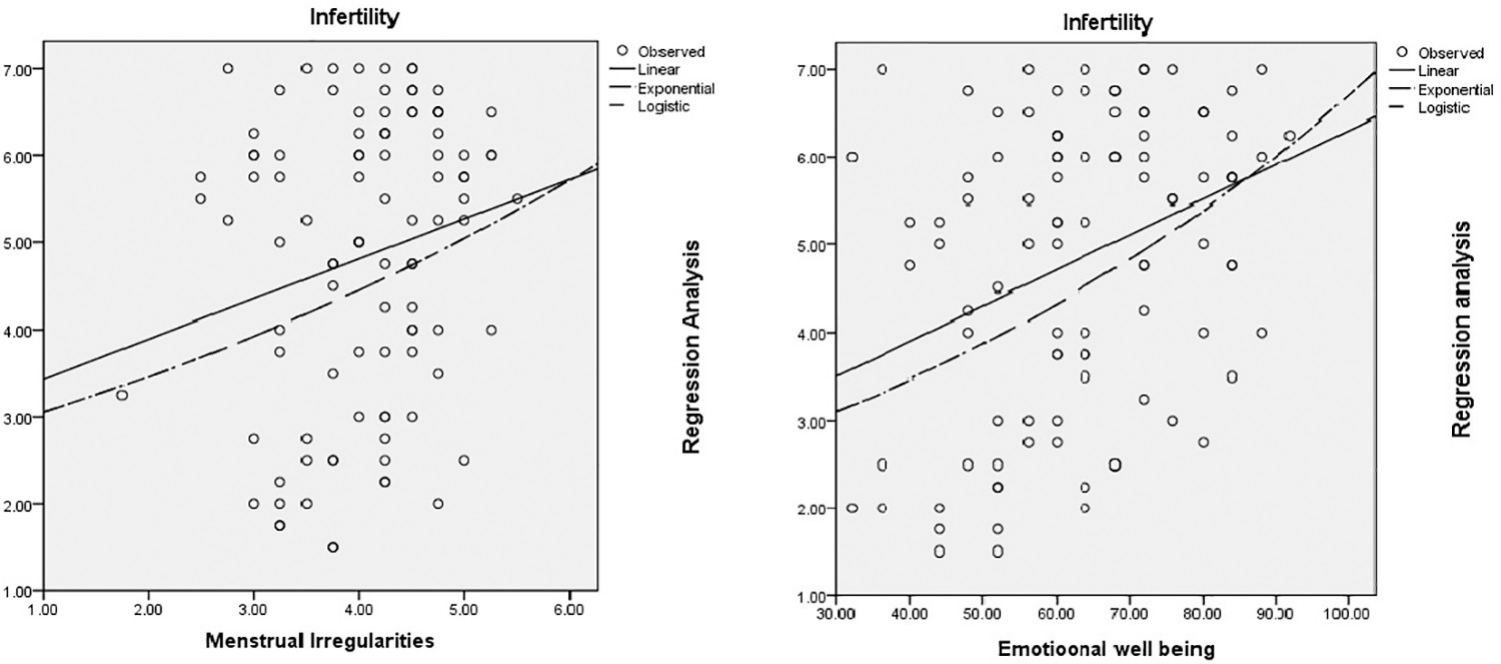


The Fig 1 and Table 8 exhibits the regression analysis data plot and we was observed strong association between infertility and menstrual irregularities (P<0.049) as well as emotional well being (P<0.001) of PCOS patients. We also observed infertility (P<0.001) and hirsutism (P<0.05) as a major predictor affecting in all domain scores (Table 9).

**Table 8:** Multiple linear regression analysis of possible related factors in HRQOL.

**Model Summary and Parameter Estimates (A)**							
Dependent Variable: Infertility							
Equation	Model Summary					Parameter Estimates	
	R Square	F	df1	df2	Sig.	Constant	b1
Linear	.039	3.990	1	98	.049	2.978	.458
Exponential	.046	4.701	1	98	.033	2.697	.125
Logistic	.046	4.701	1	98	.033	.371	.882
The independent variable is MI.							
**Model Summary and Parameter Estimates (B)**							
Dependent Variable: Infertility							
Equation	Model Summary					Parameter Estimates	
	R Square	F	df1	df2	Sig.	Constant	b1
Linear	.116	12.922	1	98	.001	2.300	.040
Exponential	.136	15.434	1	98	.000	2.241	.011
Logistic	.136	15.434	1	98	.000	.446	.989
The independent variable is EWB.							

EWB: Emotional well being; P value less than 0.05 is considered as significant differences between the group.

MI: Menstrual irregularities; P value less than 0.05 is considered as significant differences between the group.

**Table 9:** Multiple linear regression analysis of possible related factors in HRQOL (SF 36 Score).

	General Health	Physical function	Role limitations due to physical health	Role limitations due to emotional problem	Pain	Social function	Energy/Fatigue	Emotional well-being	R square	Adjusted R Square	P-Value
Age	β	β	β	β	β	β	β	β			
≤19	-0.299	-0.347	0.018	0.122	-0.095	-0.344	0.021	0.310	0.348	0.139	0.156
20–30	-0.186	0.163	0.059	-0.231	-0.282	0.107	-0.002	-0.077	0.172	0.034	0.294
>30	-0.107	-0.344	-0.538	-0.127	1.150	0.589	-1.083	-0.116	1.00	-------	--------
**BMI**											
<18.5	-0.200	0.168	-0.875	1.074	-0.318	0.159	-0.683	1.155	0.856	0.279	0.463
18.5-<25	0.065	-0.112	-0.090	0.200	0.219	-0.170	-0.140	0.177	0.098	-0.097	0.848
25-<30	-0.232	-0.324	-0.381	0.160	0.462	0.347	-0.478	0.314	0.376	0.098	0.280
≥30	-0.169	-0.356	-0.254	-0.032	0.386	0.274	1.199	-0.752	0.519	-0.031	0.537
**Infertility**	0.254	0.062	-0.023	-0.050	0.149	0.241	0.000	0.032	0.257	0.192	0.001*
**Menstrual irregularities**	0.209	0.121	-0.081	-0.109	0.156	0.128	0.067	-0.159	0.122	.044	0.144
**Hirsutism**	0.049	0.178	-0.066	-0.255	-0.057	0.299	0.078	-0.004	0.155	0.081	0.045*

HRQOL: Health related quality of life

BMI: Body mass index

β: Beta coefficients

R: Regression

*P value less than 0.05 is considered as significant change.

## Discussion

PCOS has no any constant treatment due to its multifaceted features. However, lifestyle modification, hormonal contraceptives and some other drugs like inositol, clomiphene, eflornithine, finasteride, flutamide, letrozole, metformin, spironolactone has been reported to ameliorate the PCOS symptoms [Williams T, Mortada R, Porter S. Diagnosis and treatment of polycystic ovary syndrome. American family physician. 2016; 94(2): 106–113; Lagana AS, Garzon S, Casarin J, Franchi M, Ghezzi F. Inositol in Polycystic Ovary Syndrome: Restoring Fertility through a Pathophysiology-Based Approach. Trends Endocrinol Metab. 2018;29(11):768–780; Lagana AS, Garzon S, Unfer V. New clinical targets of d-chiro-inositol: rationale and potential applications. Expert Opin Drug Metab Toxicol. 2020;16(8):703–710].

PCOS is an endocrine disorder and its long term complications affect various aspects of HRQOL in women [23,24]. Despite the various evidence about compromised HRQOL in women with PCOS, we further explored the other determinants that may help the clinician in care of the patient well-being [17,25,26]. Overall, we demonstrated the drastically compromised HRQOL in young women suffering from PCOS. As earlier reported, the woman with PCOS belongs to a lesser age group in relation to HC indicating a higher prevalence of PCOS cases in young age woman especially in adolescents [27]. As per SDS is concerned, the major difference between PCOS and control cases was observed in case of age, BMI and level of education [28]. Thus, all this indicated that PCOS affects HRQOL more in the young woman and the SDS definitely affect the prevalence of PCOS. The age of menarche in the majority of women was > 18 years as earlier reported [29]. This was in contrast to other reports [30]. Consistently, increased age of menarche was observed in PCOS cases indicating the impact of first menstruation in young women life and in the development of PCOS and another reproductive as well as metabolic disorder [31,32].

As previously reported, we observed a direct correlation between the PCOS and irregular or delayed menses and having no children that can be taken as a symptom of PCOS diagnosis [33]. The higher number of women having lesser than two children and lesser number of times get pregnant further supported this compromised HRQOL [30]. This compromised HRQOL in PCOS case was further supported by our study.

The overall decreased mean of BMI and age at menarche indicated as PCOS symptoms. Concurrently, mean age, age at marriage, number of children and frequency of pregnancy was less in PCOS cases than control. Consistent to the previous study, these indices corroborate above findings and strongly indicated the deterioration of HRQOL in PCOS women [28]. Altogether, higher fertility disorder in PCOS cases was observed that directly affects their HRQOL due to physical, social as well as emotional issues.

Furthermore, in PCOS cases, physical, social and emotional well-being more affected as evidenced from all eight domains of SF-36 indicating strongly compromised HRQOL than HC cases [34]. In particular, we also compare the mean score in relation to age, BMI, educational level and marital status. Consistent with the previous report, increasing the age had a more negative impact on different domains of SF-36 in PCOS cases than HC cases. Comparable scores in PCOS women with increasing age for physical health, energy and emotional well-being may be due to improved regular menses with age concurrent to improved PCOS features and loss of societal fear. Whereas, in HC cases, changes in normal life trend in the prospect of HRQOL was seen with increasing age indicating normal HRQOL [35].

In a similar fashion, with the increase in the BMI, physical activity was not affected in PCOS cases as observed from different domains of SF-36 but the emotional problem was more affected in PCOS cases in comparison to HC. This may be a major reason for compromised HRQOL in PCOS women [36,37]. In HC cases, none of the scores of SF-36 domains was different between BMI groups. Consistent to previous studies, with increasing the level of education, all the domains of SF-36 in PCOS cases have improved HRQOL. Similar to other HRQOL studies in different diseases, where well-qualified patients have better HRQOL than illiterate cases [38]. We also observed that well-qualified group probably have higher number of PCOS cases that directly support that improved SDS is a major contributing factor in developing PCOS. The differences in scores of all the domains of SF-36 were observed in married PCOS cases in contrast to unmarried. Thus, consistent to the previous report, the HRQOL of the unmarried cases was better in comparison to married women [39]. Whereas, in HC cases, all physical, social, as well as emotional wellness, were similar in both married and unmarried women. This may be due to their social independency and quality of education in young women with PCOS.

As reported earlier, infertility and hirsutism emerges as the major problem affecting the overall HRQOL and a strong association has been observed between infertility and emotional well-being [40].

## Conclusion

Our data compares the relations between PCOS and HC cases of overall HRQOL. We explored the strong association between PCOS and SES, and suggest that with increasing age and BMI PCOS patients had lower scores on SF-36; opposite association was with education level. However, Infertility emerges as the major predictor affecting overall HRQOL in PCOS cases. The present study does have its limitations of not measuring biochemical assessment and ultrasonography indices.

## References

[1] Balen AH. Polycystic ovary syndrome (PCOS). The Obstetrician & Gynaecologist. 2017;19(2):119–29.

[2] Escobar-Morreale HF. Polycystic ovary syndrome: definition, aetiology, diagnosis and treatment. Nature Reviews Endocrinology. 2018;14(5):270. 10.1038/nrendo.2018.24 29569621

[3] Centers for disease control and prevention, 2019. Accessed on 06/11/2019. https://www.cdc.gov/diabetes/library/spotlights/pcos.html.

[4] Cooney LG, Dokras A. Beyond fertility: polycystic ovary syndrome and long-term health. Fertility and sterility. 2018;110(5):794–809. 10.1016/j.fertnstert.2018.08.021 30316414

[5] Kalra P, Bansal B, Nag P, Singh JK, Gupta RK, Kumar S, et al. Abdominal fat distribution and insulin resistance in Indian women with polycystic ovarian syndrome. Fertility and sterility. 2009;91(4):1437–40. 10.1016/j.fertnstert.2008.06.037 18722605

[6] Khomami MB, Tehrani FR, Hashemi S, Farahmand M, Azizi F. Of PCOS symptoms, hirsutism has the most significant impact on the quality of life of Iranian women. PLoS One. 2015;10(4):e0123608. 10.1371/journal.pone.0123608 25874409 PMC4398498

[7] March WA, Moore VM, Willson KJ, Phillips DI, Norman RJ, Davies MJ. The prevalence of polycystic ovary syndrome in a community sample assessed under contrasting diagnostic criteria. Human reproduction. 2010;25(2):544–51. 10.1093/humrep/dep399 19910321

[8] Li Y, Li Y, Ng EH, Stener-Victorin E, Hou L, Wu T, et al. Polycystic ovary syndrome is associated with negatively variable impacts on domains of health-related quality of life: evidence from a meta-analysis. Fertility and sterility. 2011;96(2):452–8. 10.1016/j.fertnstert.2011.05.072 21703610

[9] Kumarapeli VL, Seneviratne RD, Wijeyaratne CN. Health‐related quality of life and psychological distress in polycystic ovary syndrome: a hidden facet in South Asian women. BJOG: An International Journal of Obstetrics & Gynaecology. 2011;118(3):319–28. 10.1111/j.1471-0528.2010.02799.x 21134104

[10] Brady C, Mousa SS, Mousa SA. Polycystic ovary syndrome and its impact on women’s quality of life: More than just an endocrine disorder. Drug, healthcare and patient safety. 2009;1:9. 10.2147/dhps.s4388 21701605 PMC3108690

[11] Yii MF, Lim CE, Luo X, Wong WS, Cheng NC, Zhan X. Polycystic ovarian syndrome in adolescence. Gynecological Endocrinology. 2009;25(10):634–9. 10.1080/09513590903015551 19533479

[12] Micucci C, Valli D, Matacchione G, Catalano A. Current perspectives between metabolic syndrome and cancer. Oncotarget. 2016;7(25):38959. 10.18632/oncotarget.8341 27029038 PMC5122443

[13] Bradley C. Measuring quality of life in diabetes. Diabetes Annual. 1996;10(1):207–24.

[14] Mahalingaiah S, Diamanti-Kandarakis E. Targets to treat metabolic syndrome in polycystic ovary syndrome. Expert opinion on therapeutic targets. 2015;19(11):1561–74. 10.1517/14728222.2015.1101067 26488852 PMC4883581

[15] Karimi M, Brazier J. Health, health-related quality of life, and quality of life: what is the difference?. Pharmacoeconomics. 2016;34(7):645–9. 10.1007/s40273-016-0389-9 26892973

[16] Jones GL, Hall JM, Lashen HL, Balen AH, Ledger WL. Health‐related quality of life among adolescents with polycystic ovary syndrome. Journal of Obstetric, Gynecologic & Neonatal Nursing. 2011;40(5):577–88. 10.1111/j.1552-6909.2011.01279.x 22273414

[17] Kaczmarek C, Haller DM, Yaron M. Health-related quality of life in adolescents and young adults with polycystic ovary syndrome: a systematic review. Journal of pediatric and adolescent gynecology. 2016;29(6):551–7. 10.1016/j.jpag.2016.05.006 27262833

[18] ESHRE TR ASRM-Sponsored PCOS Consensus Workshop Group. Revised 2003 consensus on diagnostic criteria and long-term health risks related to polycystic ovary syndrome. Fertility and sterility. 2004;81(1):19–25. 10.1016/j.fertnstert.2003.10.004 14711538

[19] Angin P, Yoldemir T, Atasayan K. Quality of life among infertile PCOS patients. Archives of gynecology and obstetrics. 2019;300(2):461–7. 10.1007/s00404-019-05202-z 31172306

[20] Ware JE Jr, Kosinski M, Gandek B, Aaronson NK, Apolone G, Bech P, et al. The factor structure of the SF-36 Health Survey in 10 countries: Results from the IQOLA Project. Journal of clinical epidemiology. 1998;51(11):1159–65. 10.1016/s0895-4356(98)00107-3 9817133

[21] Brazier JE, Harper R, Jones NM, O’cathain A, Thomas KJ, Usherwood T, et al. Validating the SF-36 health survey questionnaire: new outcome measure for primary care. British medical journal. 1992;305(6846):160–4. 10.1136/bmj.305.6846.160 1285753 PMC1883187

[22] Bottcher B, Fessler S, Friedl F, Toth B, Walter MH, Wildt L, et al. Health-related quality of life in patients with polycystic ovary syndrome: validation of the German PCOSQ-G. Archives of gynecology and obstetrics. 2018;297(4):1027–35. 10.1007/s00404-017-4623-2 29249009 PMC5849657

[23] Barthelmess EK, Naz RK. Polycystic ovary syndrome: current status and future perspective. Frontiers in bioscience (Elite edition). 2014;6:104. 10.2741/e695 24389146 PMC4341818

[24] Barnard L, Ferriday D, Guenther N, Strauss B, Balen AH, Dye L. Quality of life and psychological well being in polycystic ovary syndrome. Human reproduction. 2007;22(8):2279–86. 10.1093/humrep/dem108 17537782

[25] Teede H, Deeks A, Moran L. Polycystic ovary syndrome: a complex condition with psychological, reproductive and metabolic manifestations that impacts on health across the lifespan. BMC medicine. 2010;8(1):41. 10.1186/1741-7015-8-41 20591140 PMC2909929

[26] McCook JG, Reame NE, Thatcher SS. Health‐related quality of life issues in women with polycystic ovary syndrome. Journal of Obstetric, Gynecologic, & Neonatal Nursing. 2005;34(1):12–20. 10.1177/0884217504272945 15673641

[27] Rowlands IJ, Teede H, Lucke J, Dobson AJ, Mishra GD. Young women’s psychological distress after a diagnosis of polycystic ovary syndrome or endometriosis. Human Reproduction. 2016;31(9):2072–81. 10.1093/humrep/dew174 27412249

[28] Rzońca E, Bień A, Wdowiak A, Szymański R, Iwanowicz-Palus G. Determinants of quality of life and satisfaction with life in women with polycystic ovary syndrome. International journal of environmental research and public health. 2018;15(2):376. 10.3390/ijerph15020376 29470449 PMC5858445

[29] Carroll J, Saxena R, Welt CK. Environmental and genetic factors influence age at menarche in women with polycystic ovary syndrome. Journal of Pediatric Endocrinology and Metabolism. 2012;25(5–6):459–66. 10.1515/jpem-2012-0047 22876539 PMC3597236

[30] Okamura Y, Saito F, Takaishi K, Motohara T, Honda R, Ohba T, et al. Polycystic ovary syndrome: early diagnosis and intervention are necessary for fertility preservation in young women with endometrial cancer under 35 years of age. Reproductive medicine and biology. 2017;16(1):67–71. 10.1002/rmb2.12012 29259453 PMC5715875

[31] Welt CK, Carmina E. Lifecycle of polycystic ovary syndrome (PCOS): from in utero to menopause. The Journal of Clinical Endocrinology & Metabolism. 2013;98(12):4629–38. 10.1210/jc.2013-2375 24064685 PMC3849665

[32] Sadrzadeh S, Painter RC, Lambalk CB. Developmental origins of polycystic ovary syndrome (PCOS), a case control study comparing birth weight in women with PCOS and control group. Gynecological Endocrinology. 2016;32(10):856–9. 10.1080/09513590.2016.1186632 27222928

[33] Williams T, Mortada R, Porter S. Diagnosis and treatment of polycystic ovary syndrome. American family physician. 2016;94(2):106–13. 27419327

[34] Moghadam ZB, Fereidooni B, Saffari M, Montazeri A. Measures of health-related quality of life in PCOS women: a systematic review. International journal of women’s health. 2018;10:397. 10.2147/IJWH.S165794 30123008 PMC6078086

[35] Shishehgar F, Ramezani Tehrani F, Mirmiran P, Hajian S, Baghestani AR. Comparison of the association of excess weight on health related quality of life of women with polycystic ovary syndrome: an age-and BMI-matched case control study. PloS one. 2016;11(10):e0162911. 10.1371/journal.pone.0162911 27736861 PMC5063389

[36] Milone M, De Placido G, Musella M, Fernandez LM, Fernandez LV, Campana G, et al. Incidence of successful pregnancy after weight loss interventions in infertile women: a systematic review and meta-analysis of the literature. Obesity surgery. 2016;26(2):443–51. 10.1007/s11695-015-1998-7 26661108

[37] Panico A, Messina G, Lupoli GA, Lupoli R, Cacciapuoti M, Moscatelli F, et al. Quality of life in overweight (obese) and normal-weight women with polycystic ovary syndrome. Patient preference and adherence. 2017;11:423. 10.2147/PPA.S119180 28280314 PMC5338969

[38] Di Fede G, Mansueto P, Longo RA, Rini G, Carmina E. Influence of sociocultural factors on the ovulatory status of polycystic ovary syndrome. Fertility and sterility. 2009;91(5):1853–6. 10.1016/j.fertnstert.2008.02.161 18455164

[39] Lamb JD, Johnstone EB, Rousseau JA, Jones CL, Pasch LA, Cedars MI, et al. Physical activity in women with polycystic ovary syndrome: prevalence, predictors, and positive health associations. American journal of obstetrics and gynecology. 2011;204(4):352–e1. 10.1016/j.ajog.2010.12.006 21288501

[40] Moghadam ZB, Fereidooni B, Saffari M, Montazeri A. Polycystic ovary syndrome and its impact on Iranian women’s quality of life: a population-based study. BMC Womens Health. 2018;11;18(1):164. 10.1186/s12905-018-0658-1 30305063 PMC6180458

